# Unveiling the Ovarian Cell Characteristics and Molecular Mechanism of Prolificacy in Goats via Single-Nucleus Transcriptomics Data Analysis

**DOI:** 10.3390/cimb46030147

**Published:** 2024-03-11

**Authors:** Sanbao Zhang, Yirong Wei, Xiaotong Gao, Ying Song, Yanna Huang, Qinyang Jiang

**Affiliations:** 1 College of Animal Science and Technology, Guangxi University, Nanning 530004, China; gxuzsb@126.com (S.Z.); 15778409620@163.com (Y.W.); gaoxt855@163.com (X.G.); sy18848861569@163.com (Y.S.); 2 State Key Laboratory for Conservation and Utilization of Subtropical Agro-Bioresources, Animal Reproduction Institute, Guangxi University, Nanning 530004, China

**Keywords:** snRNA-seq, goat, ovary, cell-type, prolificacy, granulosa cells

## Abstract

Increases in litter size, which are influenced by ovulation, are responsible for between 74% and 96% of the economic value of genetic progress, which influences selection. For the selection and breeding of highly prolific goats, genetic mechanisms underlying variations in litter size should be elucidated. Here, we used single-nucleus RNA sequencing to analyze 44,605 single nuclei from the ovaries of polytocous and monotocous goats during the follicular phase. Utilizing known reference marker genes, we identified 10 ovarian cell types characterized by distinct gene expression profiles, transcription factor networks, and reciprocal interaction signatures. An in-depth analysis of the granulosa cells revealed three subtypes exhibiting distinct gene expression patterns and dynamic regulatory mechanisms. Further investigation of cell-type-specific prolificacy-associated transcriptional changes elucidated that “downregulation of apoptosis”, “increased anabolism”, and “upstream responsiveness to hormonal stimulation” are associated with prolificacy. This study provides a comprehensive understanding of the cell-type-specific mechanisms and regulatory networks in the goat ovary, providing insights into the molecular mechanisms underlying goat prolificacy. These findings establish a vital foundation for furthering understanding of the molecular mechanisms governing folliculogenesis and for improving the litter size in goats via molecular design breeding.

## 1. Introduction

In mammals, reproduction is an important and complex biological process. From a physiological perspective, the reproductive performance of female animals is determined by a series of biological events that occur from egg maturation to birth [1]. An increased number of offspring is largely associated with increased ovulation rates during the estrous cycle [2,3,4]. Certain mammals (including humans) often yield more than one offspring per parity. Within the realm of livestock, low fecundity (i.e., litter size) is a major limitation in the development of animal husbandry and genetics.

The ovary is a complex structure consisting of numerous heterogeneous cell types, including follicles in various stages of development. The ovarian follicle is the fundamental functional unit within the ovary, composed of oocytes, surrounding granulosa cells (GCs), and/or theca cells [5]. Follicular development is a highly coordinated process. Follicle cyclic recruitment, spatial displacement, follicle atresia, and ovulation are implicated events resulting from the release of molecular signals by somatic cells [6]. Each primordial follicle has the potential to undergo folliculogenesis and develop into a primary follicle, then into a secondary follicle, and ultimately into a mature follicle capable of ovulation or degeneration, akin to the majority of follicles that do not undergo maturation [7,8]. From the initial 1 million follicles present in the mammalian ovary at birth, only a mere 500 follicles progress to the ovulatory phase, while the remaining follicles undergo degeneration [9]. However, the molecular mechanisms regulating oocyte maturation, follicular growth, and regression, which contribute to the maintenance of ovarian tissue homeostasis and the number of ovulations during the estrous cycle, remain unclear [10]. Additionally, our understanding of the communication among ovarian niches, signaling pathways, critical events, and specific transcription factors (TFs) that regulate various ovarian cell types remains limited.

The Nubian goat, native to Africa, exhibits good adaptability and reproductive performance, with more than two litters per parity. In southwest China, they have been groomed on a large scale to improve the quality of local goat population since their introduction in about 1985. Du’an goats, exhibiting almost no gene communication of species or groups of species and artificial imprinting for commercial characters, are a geographical indication of the agricultural product of Du’an Yao Autonomous County, Hechi, Guangxi Zhuang Autonomous Region, China, and the litter size is small (mostly one litter per parity). They are considered an ideal biological model for investigating the mechanism governing ovarian follicle development, ovulation, and follicular atresia [11]. In addition, the molecular mechanism underlying the significant difference in litter size between Du’an and Nubian goats remains unclear.

In the context of a heterogeneous organ such as the ovary, conventional bulk RNA-sequencing (RNA-seq) approaches fail to accurately reveal cell-type-specific changes in gene expression profiles, particularly for rare cell types. The single-cell/nuclei RNA sequencing (sc/nRNA-seq) technique can efficiently identify cell types, uncover heterogeneity, and construct developmental trajectories. Studies have demonstrated the ability of sc/nRNA-seq to determine the molecular underpinnings of dominant follicle selection, follicular atresia, and prolificacy in mammals [12,13]. Therefore, in this study, we utilized snRNA-seq data from the ovaries of polytocous and monotocous goats to reveal distinctive characteristics of ovarian cells and the molecular mechanisms governing prolificacy in goats. Our study establishes a vital foundation for furthering understanding of the folliculogenesis molecular mechanisms in mammals.

## 2. Materials and Methods

### 2.1. Animal Preparation and Animal Tissue Collection

The snRNA-seq data utilized in this study were derived from previously collected data by our research team. Briefly, the Nubian and Du’an goats were obtained from the Standardized Breeding Farm in Guangxi Province in this study, and their litter sizes were recorded. Between 2016 and 2020, Du’an goats exhibited a range of 1–2 lambs per parity, with an average litter size of 1.28. In contrast, Nubian goats had an average litter size of 2.13, approximately 1–5 lambs per parity. The experimental group comprised two Nubian goats in the polytocous group (litter size = 3, about 3 years old, and 3 births) and two Du’an goats in the monotocous group (litter size = 1, about 3 years old, and 3 births) based on three generations of lamb breeding records. These goats were subjected to estrous synchronization through controlled internal drug release (progesterone 300 mg, InterAg, Hanilton, New Zealand) for 17 days to achieve a synchronized estrus phase. Subsequently, two Nubian and two Du’an goats were anesthetized and euthanized, and their ovaries were collected for snRNA-seq analysis.

### 2.2. Single-Cell RNA-Seq Library Construction and Sequencing

The left ovaries of the two Nubian and two Du’an goats were isolated 12 h after the onset of the estrus phase. After removal of the ovarian adventitia and surrounding adipose tissue, the ovarian tissues were dissociated to obtain single nuclei, and Trypan Blue staining was performed to confirm nuclear lysis. Later, single-nuclei RNA sequencing (snRNA-seq) was performed using the 10× Genomics platform on the single nuclei. Briefly, nuclei preparations and sorting single-cell nuclei were prepared using a sodium citrate lysis buffer containing Triton X-100 detergent. Subsequently, scRNA-seq libraries were constructed employing 10× Genomics Chromium Next GEM Single Cell 3′ Reagent Kits v3.1 (1000268), following a previously described protocol [14]. These libraries were subjected to quality assessments (Fragment Analyzer 2100, Agilent Technologies, Waldbronn, Germany) and sequenced (Platform: Illumina Nova 6000, San Diego, CA, USA; read length: 150 bp, paired-end). All mRNAs were randomly appended to unique molecular identifiers (UMIs) after reverse transcription, and mRNAs were quantified based on the UMIs. It was almost impossible to connect mRNA to the same UMI, which can be used to avoid the bias caused by PCR. The sequencing and subsequent bioinformatics analyses were performed by OE Biotech Co., Ltd. (Shanghai, China).

### 2.3. Data Processing and Downstream Analysis

Transcripts were mapped to the corresponding reference genome (https://www.ncbi.nlm.nih.gov/genome/10731?genome_assembly_id=281266; accessed on 12 December 2021) using the 10× Genomics Cell Ranger pipeline (v5.0.0). Subsequently, read count matrices were generated for each sample through the Cell Ranger count. These count data were then imported into Seurat R package v8 [15,16]. Quality control (QC) was performed on each library to eliminate outlier cells and genes. Assuming a Gaussian distribution of UMI/gene counts per cell, we employed a criterion to filter and exclude cells whose UMI/gene counts exceeded beyond the mean ± two-fold standard deviation (SD). Following a visual inspection of the distribution of cells based on the fraction of expressed mitochondrial genes, low-quality cells, where >20% of the count was attributed to mitochondrial genes, were excluded. Furthermore, genes expressed by at least five cells were retained. To mitigate potential biases arising from doublets, we set the threshold for cells captured during sequencing at 10%. Doublet cells were detected by employing Single-Cell Remover of Doublets (Scrublet) software (version 0.2.1) [17]. In total, 44,605 single nuclei met the QC criteria and were subsequently included in the ensuing analysis. To achieve normalized counts, we performed library size normalization in Seurat on the filtered matrix, utilizing the Log Normalize method, and the inherent variation attributed to mitochondrial gene expression was regressed. For cell clustering, principal component analysis was performed on highly variable genes, as determined by the FindVariableGenes function. In total, 17 unsupervised cell clusters were derived. The clustering results for individual or grouped samples were visualized using t-SNE. The categorization of cell types relied on the differential expression of cluster-specific marker genes, as identified using the Findmarkers function. Gene Ontology (GO) enrichment analysis of cell-type-specific marker genes was performed using Metascape (http://metascape.org/gp/index.html#/main/step1; accessed on 22 February 2022) online tools. Representative GO terms for each cell type were selected from the top 20 terms with a significance threshold of *p* < 0.05.

### 2.4. Weighted Gene Co-Expression Network Analysis (WGCNA)

WGCNA was performed on pseudo-cells using the WGCNA R package (v1.69) [18]. From the original dataset, 4000 genes and 25,000 nuclear samples were filtered out based on an SD threshold of ≤0.01. Consequently, 3944 genes and 25,000 samples were retained for subsequent analysis. The top 4000 differentially expressed genes (DEGs) determined by Seurat were analyzed. Briefly, to construct a weighted co-expression network model, we constructed a topological overlap matrix by varying the soft power from 1 to 30. The hub genes in each module were identified as module eigengenes. GO enrichment analysis was performed using the ClusterProfiler R package with the hub gene datasets, and the BH method was employed for multiple test corrections. GO terms with a *p* ≤ 0.05 were considered significantly enriched.

### 2.5. Identification of TFs by SCENIC

SCENIC analysis was conducted using the motifs database of RcisTarget and GRNboost (SCENIC [v1.1.2.2] [19], RcisTarget [v1.2.1], and AUCell [v1.4.1]) to identify active TFs across various ovarian cell types. Briefly, the potential target genes associated with each TF were determined through co-expression and motif analysis. The AUCell package was employed to score the activity of regulons in each cell. To assess cell-type-specific regulation, we calculated the regulator-specific score based on Jensen Shannon divergence [16]. Furthermore, the connection specificity index (CSI) for all regulators was calculated using the scFunctions package (https://github.com/FloWuenne/scFunctions/; accessed on 2 April 2022).

### 2.6. Pesudotemperal Trajectory Analysis

Pseudotime analysis of the GC populations was performed using the Monocle2 (v2.9.0) R package [20]. Genes were ordered based on genes that were expressed in at least 10 cells, exhibiting differential expression across clusters, and dispersed with a q-value < 0.01. The pseudotime trajectory’s structure was visualized utilizing the DDRTree dimensionality reduction algorithm. Briefly, the Monocle2 package was used to explore the developmental pseudotime. Raw counts in Seurat object were first converted to a CellDataSet object using the importCDS function in Monocle2. Subsequently, genes informative for ordering cells along the pseudotime trajectory were identified using the differentialGeneTest function (q-value *<* 0.01). A dimensional reduction clustering analysis was performed using the reduceDimension function, followed by trajectory inference employing the *orderCells* function with default parameters. The plot_genes_in_pseudotime function was applied to monitor gene expression changes over pseudotime. To determine the order of gene expression and functional events during cell state transitions at a single-cell resolution, we conducted GeneSwitches analyses. Specifically, gene expression data were first binarized into 1 (on) or 0 (off) using the binarize_exp function in the GeneSwitches package (fix_cutoff = TRUE, binarize_cutoff = 0.05). Gaussian noise, with a mean of 0 and s.d. of 0.1, was introduced to the gene expression data to ensure numerical stability in the model fitting. Genes lacking a distinct bimodal “on–off” distribution were filtered out. Subsequently, logistic regression was fitted to model the binary states (on or off) using the find_switch_logistic_fastglm function, with the random downsampling of zero expressions (downsample = TRUE) to account for genes with high zero inflation. Finally, the top 50 best-fitting genes (high McFadden’s Pseudo R^2^) were visualized along the pseudotimeline, with “switched on” genes depicted above the line and “switched off” genes depicted below the line.

### 2.7. Analysis of Cell–Cell Communication

CellPhoneDB (v2.0, https://github.com/Teichlab/cellphonedb; accessed on 15 June 2022) [21] was employed to systematically predict cell–cell communications based on ligand–receptor analysis, utilizing default parameters. Receptors or ligands expressed in at least 10% of cells of a particular type, meeting the significance threshold of *p* < 0.05, were subsequently analyzed. The Igraph software (Version 1.0.1) package and the Circular Visualization R package were utilized to visualize interaction links for the selected significant receptor–ligand pairs.

### 2.8. Identification DEGs

To identify prolificacy-associated DEGs between Nubian and Du’an goat individuals in each specific cell type, we employed the FindMarkers function in the R package Seurat, based on a *t*-test [16]. *p*-value < 0.05 and |log_2_foldchange| > 0.58 were set as the threshold for significant differential expression.

### 2.9. Gene Enrichment Analysis

Enrichment analysis of significant DEGs was performed using Metascape (http://metascape.org/gp/index.html#/main/step1; accessed on 15 April 2022). A combined score was calculated to determine enrichment ranking for pathways, ontology, TF network, and protein network analyses. This combined score was calculated using the log of the *p*-value obtained through the Fisher exact test and then multiplying it by the z-score, representing the deviation from the expected rank.

## 3. Results

### 3.1. Single-Nucleus Transcriptome Profiling of Goat Ovaries Elucidated Ovarian Cell Types and Gene Expression Signatures

**Figure 1:**
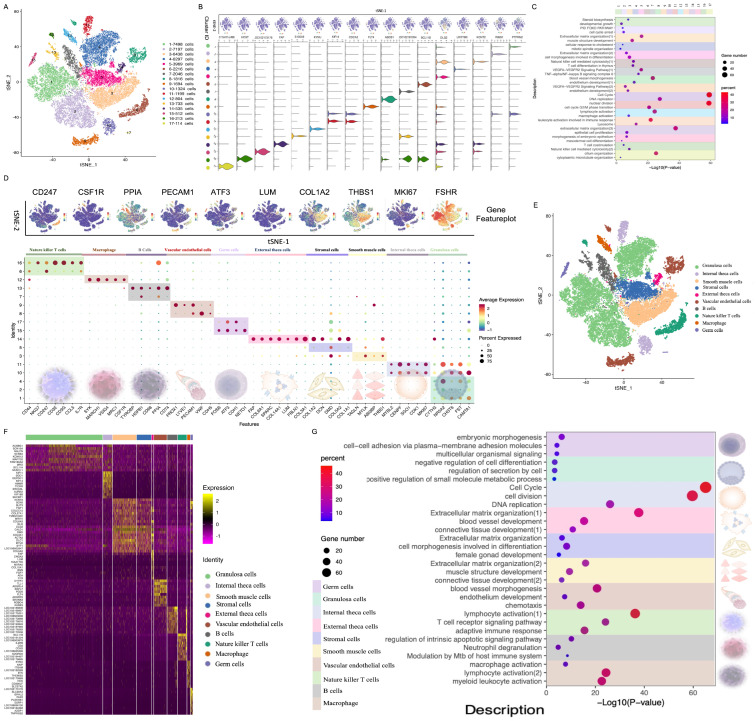
Single-nucleus transcriptomic atlas of the goat (*Capra hircus*) ovary. (**A**) t-SNE visualization of all cells, displayed with different colors for 17 distinct clusters. (**B**) Violin plots showing the expression of one representative differentially expressed gene for each cluster. (**C**) Representative Gene Ontology (GO) terms of the top 150 marker genes in each cluster. (**D**) Dot plot showing the expression of representative markers of each cell type. (**E**) t-distributed stochastic neighbor embedding (t-SNE) plot showing ten ovarian cell types. (**F**) Heatmap of the expression pattern of the top ten DEGs in each cell type. (**G**) Representative GO terms for the top 150 DEGs in each cell type.

From a total of 48,332 nuclei, an average of 54,129 reads were obtained, encompassing 5230 genes per nucleus. Raw reads were subjected to QC analysis. A high-quality transcriptome of 44,605 nuclei passed the filters and QC, averaging 1561 genes and 4049 UMIs per cell for subsequent analyses (Table S1). Upon unsupervised clustering, all ovarian cells were categorized into 17 clusters (Figure 1A). DEGs were calculated in each cluster, and filtering was performed using the following criterion: an average log_2_fold change > 0.5. These DEGs were subsequently sorted based on adjusted *p*-values (Wilcoxon rank sum test), and one representative DEG was graphed per cluster (Figure 1B). GO enrichment analyses were performed using the Metascape online tool (http://metascape.org/gp/index.html#/main/step1; accessed on 15 April 2022) to identify the biological functions of the DEGs from each cluster (Figure 1C) and facilitate cluster identification (Figure S1). To further delineate these cell clusters, we mapped the gene expression profiles of well-defined cell-type-specific markers onto a t-SNE plot, and color-coded single cells based on the expression levels of several expected marker genes (Figure 1D). GCs were located in clusters 1, 2, and 4, while the internal theca cells were primarily in clusters 10 and 11. Interstitial cells, containing smooth muscle cells, were distributed in cluster 3, and stromal cells were detected in cluster 5. Furthermore, external theca cells were observed in cluster 14, and vascular endothelial cells were primarily concentrated in clusters 8 and 9. The immune profile, including B cells, was identified in clusters 7 and 13; natural killer T cells were present in clusters 6 and 16; and macrophages were primarily found in cluster 12. Finally, germ cells were located in clusters 15 and 17. Subsequently, 10 cell types (Figure 1E) and their DEGs (Figure 1F) were identified. GO analysis of the DEGs revealed the biological functions and unique gene expression signatures/characteristics of each ovarian cell type (Figure 1G). For example, pathways enriched for GCs were “response to insulin”, “regulation of the canonical Wnt signaling pathway”, and “fatty acid metabolism”. Vascular endothelial cells exhibited enrichment in “blood vessel morphogenesis”, “hemostasis”, “cellular response to growth factor stimulus”, and “extracellular matrix organization”. In summary, we systematically identified 10 distinct ovarian cell types and their associated gene expression signatures.

### 3.2. WGCNA Revealed the Biological Functions and Hub Regulatory Networks of Ovarian Cell Types

**Figure 2:**
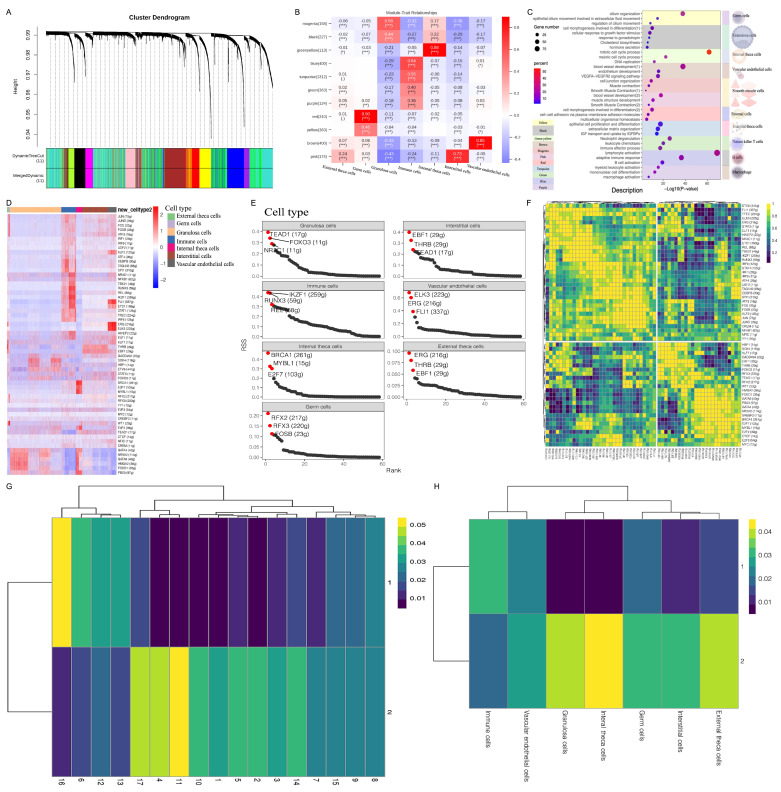
Identification of cell-type-specific hub genes and transcription factors by WGCNA and SCENIC analysis. (**A**) Dendrogram showing the gene co-expression network constructed by WGCNA. The color bar, labeled “Module colors”, beneath the dendrogram represents the module assignment of each gene. (**B**) The relationship between Modules and Seurat clusters. The upper numbers within each grid are the correlation between each module and Seurat cluster. The numbers in parentheses represent the *p*-values. * *p* < 0.05, ** *p* < 0.01 and *** *p* < 0.001. (**C**) Significantly enriched representative GO terms based on the genes in each module. (**D**) Specific distribution heatmap of regulons of all the cell types. The “regulon” refers to the regulatory network of TFs and their target genes. The numbers in parentheses next to the regulon names indicate the number of genes enriched in regulons. Abbreviation: g, gene. (**E**) Rank of the regulons in cell type based on regulon specificity score (RSS). (**F**) Heatmap for the CSI correlation clustering of the regulon module. The rows and columns represent regulons. The color changes from blue to yellow, indicating that the CSI correlation value changes from low to high. Regulons with high CSI values may have similar cellular functions and jointly regulate downstream genes. (**G**) Heatmap for the activity of the connection specificity index (CSI) module in each cluster, in accordance with Figure 1A. Rows represent the CSI modules, manually divided according to CSI clustering heatmap; columns represent different groups. The color changes from blue to yellow, indicating that the activity of the CSI module changes from low to high. The cell types corresponding to CSI modules with similar activity may have similar gene expression patterns and regulatory networks. (**H**) Heatmap for the activity of the CSI module in each cell type. Rows represent CSI modules, manually divided according to CSI clustering heatmap; columns represent different groups. The color changes from blue to yellow, indicating that the activity of the CSI module changes from low to high. The cell types corresponding to CSI modules with similar activity may have similar gene expression patterns and regulatory networks.

To systematically investigate gene dynamics, we performed a WGCNA [22]. Initially, we filtered the original dataset, consisting of 4000 genes and 25,000 nuclear samples, based on an SD threshold of ≤0.01 via Seurat QC. Finally, 3944 genes and 25,000 samples were retained through the WGCNA. Based on the selected power value (1 < power value < 30), we established a weighted co-expression network model and identified 11 gene modules (Figure 2A). Each module comprised gene sets that demonstrated a tendency to be co-expressed. Furthermore, we performed GO analyses for the genes in each module to investigate their biological functions (Figure 2C). To assign co-expressed gene functions to Seurat clusters, we constructed a correlation heatmap (Figure 2B). For example, the yellow module represented germ cells, characterized by the enriched functions of “cilium organization”, “epithelial cilium movement involved in extracellular fluid movement”, and the “regulation of cilium movement”. The genes in the black module exhibited the enriched functions “cell morphogenesis involved in differentiation”, “cellular response to growth factor stimulus”, “response to gonadotropin”, “cholesterol biosynthesis”, and “hormone secretion”, aligning with the characteristics of GCs. To understand the complex gene expression patterns in the ovary, we identified the relationships between the top 50 genes with the highest connectivity in each module, i.e., the core genes of each module. The hub genes revealed important regulatory networks in ovarian cell types (Figure S2A–K). For example, in GCs, hub genes including *CYP19A1*, *INHBA*, *INHBB*, *LHCGR*, *NR5A2*, *ROBO2*, *TMEM178B*, *TMEM200A*, and *SERPINE2* were associated with follicular development (Figure S2A). In germ cells, the hub gene *NEK5* regulates cell cycle progression during mouse oocyte maturation and preimplantation embryonic development (Figure S2K) [23]. In summary, we identified the hub regulatory networks involving diverse cell types within goat ovaries, thereby facilitating our comprehension of the reciprocal regulatory mechanisms governing ovarian cells.

### 3.3. SCENIC Analysis Revealed Key Transcription Factors (TFs) Regulating Ovarian-Specific Cell Types

**Figure 3:**
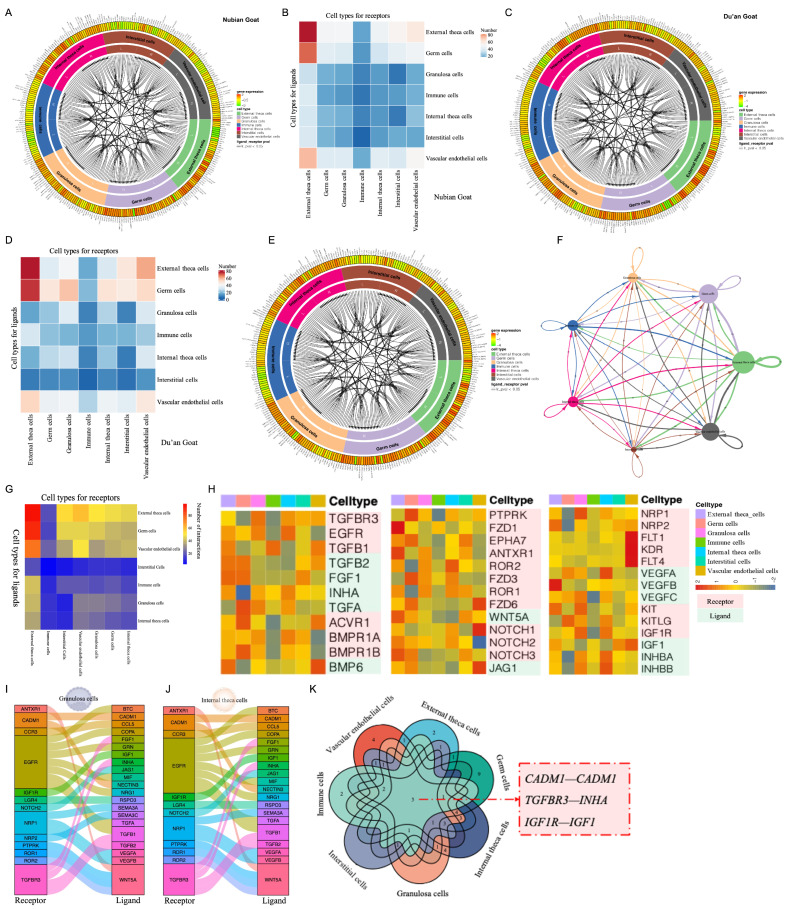
Ovary niche regulation based on ligand–receptor analysis. (**A**,**B**) Putative communications between differentially expressed receptors and ligands in the ovary and the corresponding ligands released from their niche cells of Nubian (**A**) and Du’an goats (**B**). Compartments represent cell types; their preferentially expressed receptors and ligands are labeled along the outer margin. (**C**,**D**) Heatmap of the total number of interactions between cell types of Du’an (**C**) and Nubian goats (**D**). (**E**) Putative communications between differentially expressed receptors and ligands in the ovary and the corresponding ligands released from their niche cells. Compartments represent cell types; their preferentially expressed receptors and ligands are labeled along the outer margin. (**F**) Network plots showing the ligand–receptor interaction events in each cell type with the niche cells of the ovary. Nodes are coloured by cell identity and sized by the number of interactions. Edge thickness indicates the number of interactions between the connecting cells. (**G**) Heatmap of the total number of interactions between cell types. (**H**) Gene expression signature of key signaling pathway components in goat ovary and their niche cells, including the expression of genes linked to *Wnt*, *VEGF*, *Notch*, and *TGF-β/BMP* signaling pathway. (**I**,**J**) Clues for interactions between GCs and External theca cells (**J**) and surrounding cells identified by CellphoneDB (**I**). (**K**) Venn diagram showing common receptor–ligand pairs between ovarian cells and surrounding cells. The numbers in the Venn diagram indicate the overlap numbers of receptor–ligand pairs between ovarian cells and surrounding cells.

TFs are crucial regulators of gene expression and aid in the identification of various cell types. We employed SCENIC analysis to characterize ovarian cell types [19,24]. This algorithm enables the inference of global research networks and assesses the cellular status based on scRNA data. It facilitates the scoring of gene regulatory network activity in each cell type, enabling the identification of stable cell states and their corresponding regulators. Additionally, it investigates the activity of TF regulons (i.e., TFs and their target genes). Initially, we identified modules containing genes co-expressed with TFs, followed by cis-regulatory motif analysis to identify significantly enriched modules for scoring the subnetwork activity. The results revealed 58 active regulons in the ovary (Figure 3D), with the number of target regulons ranging from 11 to 411 (Figure 3D). Additionally, we identified several TFs that modulated cell-type-specific gene regulatory networks. Notably, *NR2C1*, *FOXO3*, and *TEAD1* exhibited predominant activation in GCs, while in internal theca cells, the genes *BRCA1*, *MYBL1*, and *E2F7* were activated (Figure 3E). These TFs can serve as valuable markers for specific cell types. CSI correlation clustering of the regulon module elucidated some TFs with similar functions, providing insights into the molecular mechanisms governing follicular development and atresia (Figure 3F). For example, in the case of *IKZF1* and *RUNX3*, *RUNX3* has been demonstrated to regulate folliculogenesis and steroidogenesis in the GCs of immature mice [25], indicating that *IKZF1* may share similar functions. In the case of *HMGA2* and *FOXO1*, reports suggest that *FOXO1* regulates the follicle-stimulating hormone (FSH)-mediated inhibition of apoptosis in mouse GCs [26,27], indicating a potential role for *HMGA2* in follicular development. Furthermore, in the case of *PBX3* and *GATA4*, *GATA4*, a GC factor, regulates inhibin-α activation through the TGF-β pathway [28], implying that *PBX3* may regulate folliculogenesis via the TGF-β pathway. Furthermore, CSI correlation clustering of the regulon module within 17 clusters showed that the cluster–cluster distance exhibited consistency with Figure S1. The 10 main cell types were accurately classified (Figure 3G). Notably, our results revealed a similarity in the regulon CSI between GCs and internal theca cells (Figure 3H), suggesting the possibility of similar biological functions. This prompts the hypothesis that internal theca cells may originate from GCs.

### 3.4. Niche Regulation of Ovary Cell Type Revealed by Cell–Cell Communication

To elucidate niche regulation in the goat ovary, we attempted to explore intercellular communication among various cell types. Notably, the results revealed no significant disparity in cell–cell communication between the Nubian goat and the Du’an goat (Figure 3A–D and Figure S3A–D). We quantified interaction links between the various cell types through ligand–receptor analysis, considering the integration of the four ovarian cell types (Figure 3F,G). The immune cell ligands and interstitial cell receptors engaged with receptors and ligands of various cell types (Figure 3G). Notably, external theca cells exhibited the highest link counts in both ligands and receptors, indicating an auto- or intercellular mode of regulation (Figure 3G). In terms of the tentative ligands or receptors identified in the ovary, external theca cells, germ cells, and vascular endothelial cells influenced a substantial number of interactions.

A cell–cell interaction map was constructed by correlating ligands with their respective receptors on follicles and their associated niche cells (Figure S3E), which indicated a dual role for follicles: they can be regulated by niche cells or serve as central regulators of the surrounding niche cells (Figure 3E). Previous studies have shown that the regulation of follicular development within the niche involves multiple signaling pathways, such as RA signaling [29], BMP signaling [30], KIT signaling (KITLG) [31], PI3K/AKT signaling [32,33], TGF-β/SMAD signaling [10,34], Wnt5a/β-catenin signaling, and the Notch pathway [35]. Figure 3H illustrates the key signaling pathways expressed in the goat ovary and in the surrounding “niche cells”. Overall, the KIT, Wnt, INHB, TGF-β/BMP, and Notch signaling pathways, ligands, and receptors were expressed in a cell-type-specific manner. For instance, the ligand of the KIT signaling pathway (KITLG) was explicitly expressed by GCs, while the receptor was abundantly expressed in the follicular GCs, playing a crucial role in germ cell survival [31]. Additionally, receptors such as TGFBR3, TGFB1, ACVR1, and BMPR2, along with their corresponding ligands (including FGF1, FGFB1, INHA, and TGFB2), were expressed by various ovarian cell types, indicating both intra- and inter-cellular communication mediated by the TGF-β/BMP signaling pathway (Figure 3H). Similarly, the Wnt/β-catenin pathway ligand Wnt5A was expressed by each ovarian cell type, while its corresponding receptors were expressed in GCs, germ cells, vascular endothelial cells, and external and internal theca cells (Figure 3H). IGF1-IGF1R expression was notably high in GCs, germ cells, vascular endothelial cells, and external and internal theca cells (Figure 3H). Previous studies have shown that KIT plays an indispensable role in both primordial follicle activation and folliculogenesis, and its expression is enhanced by IGF-1 via its receptor, IGF-1R [31]. We identified VEGFA, VEGFB, and VEGFC ligands on each cell type of the ovary, along with their corresponding receptors, including NRP1, NRP2, FLT1, KDR, and FLT4 (Figure 3H). VEGFA influences the growth of the follicles by regulating the balance between pro-angiogenesis and anti-angiogenesis [36]. As shown in Figure 3H, Notch1/2/3-JAG1 ligands were primarily expressed in GCs, germ cells, and vascular endothelial cells, as well as external and internal theca cells. In addition, the constructed cell niche network revealed a shared receptor–ligand relationship between GCs and internal theca cells (Figure 3I,J), suggesting a similarity in their biological functions within the ovary. Furthermore, certain receptor–ligand pairs, including CADM1–CADM1, TGFBR3–INHA, and IGF1R–IGF1, have emerged as key players in primordial follicle activation, follicular development, and ovulation (Figure 3K and Figure S4A–E).

### 3.5. GC Subtype Identification and Transcriptional Signature Analysis of Goat Ovaries

**Figure 4:**
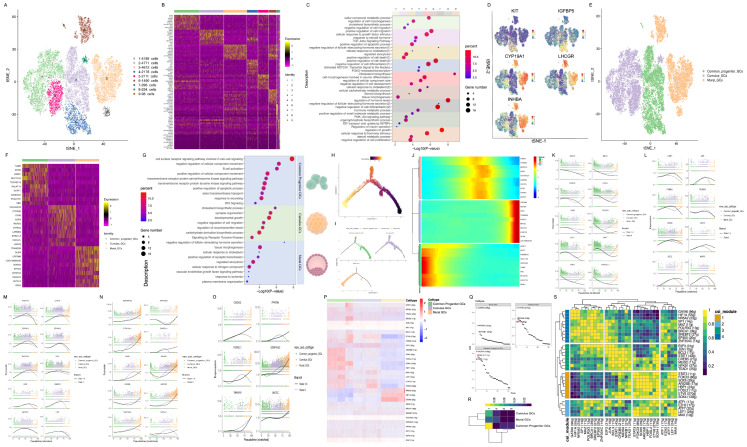
GC subtype identification and transcriptional signatures analysis of the goat ovary. (**A**) t-SNE visualization of GCs using different colors for nine distinct clusters. (**B**) Heatmap showing the expression of specific markers in each cluster. (**C**) Representative GO terms for the top 150 marker genes in each cluster. (**D**) t-SNE cluster map showing the expression of the candidate cell-type-specific markers on GCs. A gradient of blue to red indicates a low to high gene expression level. (**E**) t-SNE visualization of GCs using different colors for three subtypes. (**F**) Heatmap showing expression signatures of the top 10 specifically expressed genes in each GC subtype. (**G**) Representative GO terms of the top 150 marker genes in each GC subtype. (**H**) Cell trajectories on the pseudotime of GCs generated by Monocle 2. (**I**) Distribution of GC subtypes on the pseudotime trajectory. (**J**) Pseudo-temporal dynamics of pseudotime-dependent genes in three GC subtypes. Each row is normalized to its peak value over pseudotime. (**K**) Gene expression dynamics of representative genes of common progenitor GCs. (**L**,**M**) Gene expression dynamics of representative genes of cumulus GCs. (**N**,**O**) Gene expression dynamics of representative genes of mural GCs. (**P**) Specific distribution heatmap of regulons in three GC subtypes during GC differentiation. The “regulon” refers to the regulatory network of TFs and their target genes. The numbers in parentheses next to the regulon names indicate the number of genes enriched in the regulons. (**Q**) Rank for regulons in three GC subtypes during GC differentiation based on regulon specificity score (RSS). (**R**) Heatmap for the activity of CSI module in each GC subtype during folliculogenesis. Rows represent CSI modules manually divided according to CSI clustering heatmap; columns represent different groups. The color changes from blue to yellow, indicating that the activity of the CSI module changes from low to high. The cell types corresponding to CSI modules demonstrating similar activity may have similar gene expression patterns and regulatory networks. (**S**) Heatmap for the CSI correlation clustering of regulon modules. The rows and columns represent the regulons. The color changes from blue to yellow, indicating that the CSI correlation value changes from low to high.

To investigate GC characteristics, we analyzed the *FSHR^high^/NR5A1^high^/CAMTA1^high^/CYTH3^high^* cells again. These cells were divided into nine clusters through unsupervised clustering (Figure 4A), and the biological function of each cluster was investigated via GO enrichment analysis of DEGs (Figure 4B,C) to identify the unique differentiating characteristics of GCs. Based on the expression profile of mural GC markers, namely, CYP19A1, LHCGR, and INHBA (Figure 4D), alongside the functional enrichment analyses of each cluster, the GCs were categorized into three subtypes: common progenitor GCs, cumulus GCs, and mural GCs (Figure 4E).

The biological function of the three GC subtypes was analyzed through the GO enrichment analysis of DEGs (Figure 4F,G). The enriched functions associated with DEGs in common progenitor GCs were the “negative regulation of cellular component movement” and “positive regulation of cellular component movement”. These results suggest that all the states of the primordial follicle, encompassing both resting and activated primordial follicles that mature into primary follicles, are established in the ovary prior to birth [37]. In addition, GO terms such as the “positive regulation of apoptotic process” and “WNT signaling” were also enriched in common progenitor GCs, suggesting the potential involvement of the WNT signaling pathway in maintaining follicle numbers by regulating follicle atresia in the primordial follicle and its subsequent activation. For cumulus GCs, the enriched pathways included the “cholesterol biosynthetic process”, “negative regulation of cell migration”, and “carbohydrate derivative biosynthetic process”, indicating that cumulus GCs serve as the primary source of nutrients for regulating follicular development and maturation. In the case of mural GCs, GO analysis revealed enrichment in terms such as the “negative regulation of follicle-stimulating hormone secretion”, “cellular response to cholesterol”, “vascular endothelial growth factor signaling pathway”, and “plasma membrane organization”.

Figure 4H–J illustrate the differentiation trajectory of GCs, commencing with a common progenitor GC population and culminating in cumulus GCs. As follicular development progresses, cumulus GCs differentiate into mural GCs (Figure 4H). Figure 4K shows that genes associated with cell growth, the cell cycle, and the WNT pathway, such as *PRP16*, *CDK14*, *IGFBP5*, *CCND3*, and *ARNT2*, were abundantly expressed by the common progenitor GCs. As shown in Figure 4L,M, high levels of genes regulating cell growth and differentiation (*IGFBP2*, *CHST8*, *RERG*, *HMGCR*, and *EDA*) are evident in cumulus GCs. Furthermore, a high expression of genes associated with the regulation of hormone secretion and ovulation, such as *LHCGR*, *CYP19A1*, *INHA*, *INHBA*, *INHBB*, *SERPINE2*, and *NR5A1*, was observed in mural GCs (Figure 4N,O). A SCENIC analysis revealed 31 active regulons in GCs (Figure 4P). Approximately 11–270 TF target genes associated with these regulons were identified (Figure 4P). The results demonstrated the expression of *NR5A2*, *GATA6*, and *NFKB1* in mural GCs, *SOX4*, *FOSL2*, and *STAT3* in common progenitor GCs, and primarily *ETV6*, *NR5A2*, and *ATF7* in cumulus GCs (Figure 4Q). CSI analysis suggested that *ARID5B* may potentially serve a similar function with *Hbp1* (Figure 4R,S). In conclusion, these results elucidate the gene expression patterns and dynamic regulatory mechanisms at play within GCs.

### 3.6. Differences in Ovarian Cell Expression Profiles between the Polytocous and Monotocous Goats

**Figure 5:**
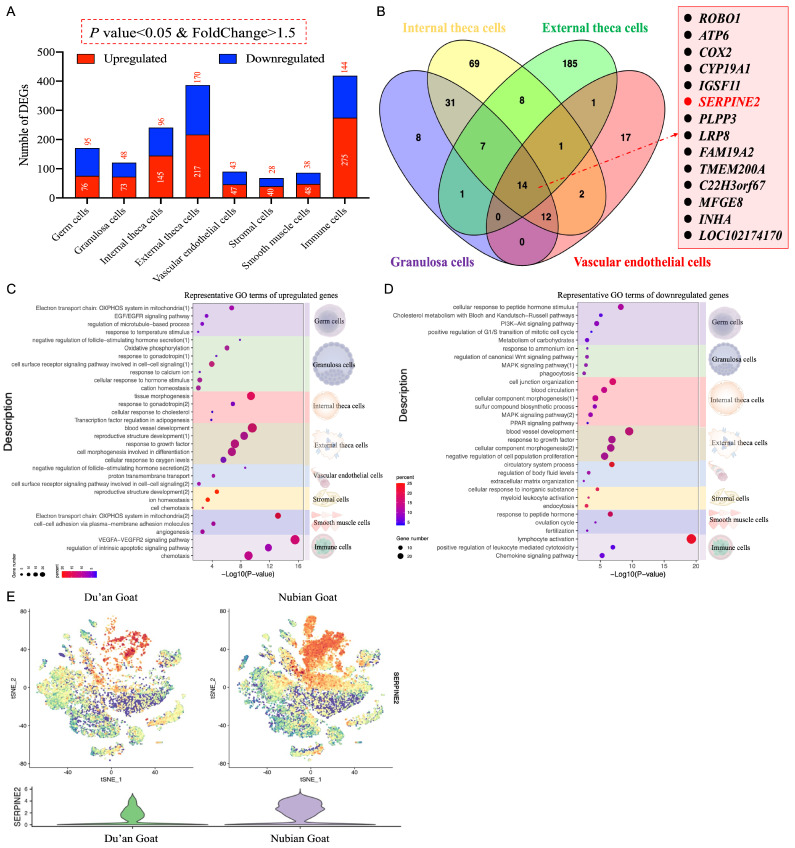
Differences in ovarian cell expression profiles between the polytocous and monotocous goats. (**A**) Histogram showing the distribution of DEGs between Nubian and Du’an goats in each cell type: red boxes correspond to upregulated DEGs; blue boxes correspond to downregulated DEGs in each cell type. (**B**) Venn diagram showing the intersection of DEGs in the internal and external theca cells, GCs, and vascular endothelial cells; the intersection of DEGs is represented on the side. (**C**) Representative GO terms of upregulated genes enriched between Nubian and Du’an goats in each cell type. (**D**) Representative GO terms of downregulated genes enriched between Nubian and Du’an goats in each cell type. (**E**) t-SNE cluster map showing *SERPINE2* expression between Nubian and Du’an goats. A gradient of blue to red indicates the low to high gene expression profile. Violin plots show the expression of the candidate cell-type-specific markers of GCs in 17 clusters.

We further explored the prolificacy-associated gene expression patterns in ovarian cells and prolificacy-associated DEGs in each cell type (Figure 5A). Most of the prolificacy–associated DEGs exhibited cell subtype specificity. Furthermore, *ROBO1*, *ATP6*, *COX2*, *CYP19A1*, *IGSF11*, *SERPINE2*, *PLPP3*, *LRP8*, *FAM19A2*, *TMEM200A*, *C22H3orf67*, *MFGE8*, *INHA*, and *LOC102174170* were upregulated and common among four distinct cell types (Figure 5B). These genes are associated with folliculogenesis, indicating a potential influence of specific cell types on prolificacy.

GO analysis was performed on DEGs in the germ cells. Upregulated genes exhibited enrichment in the “electron transport chain: OXPHOS system in mitochondria”, whereas downregulated genes were enriched in the “PI3K-KT signaling pathway” and “metabolism of carbohydrates” (Figure 5C,D).

In GCs, upregulated genes exhibited enrichment in functions associated with the “negative regulation of follicle-stimulating hormone secretion”, “oxidative phosphorylation”, “response to gonadotropin”, and “cellular response to hormone stimulus”. Furthermore, the downregulated genes in GCs were enriched in functions pertaining to the “response to regulation of the canonical WNT signaling pathway” and the “MAPK signaling pathway”. The downregulated genes were associated with WNT and MAPK signaling pathways, which play pivotal roles in GCs and follicular development (Figure 5C,D).

In internal theca cells, upregulated genes exhibited enrichment in functions including “tissue morphogenesis”, “response to gonadotropin”, and “cellular response to cholesterol”, while downregulated genes in internal theca cells were enriched in functions including “blood circulation” and the “MAPK signaling pathway”. These results were consistent with pathways enriched in GCs, thus validating the potential significance of the GnRH and MAPK signaling pathways as crucial regulators of heightened reproductive performance in Nubian goats. In addition, the upregulated genes were associated with the cholesterol response, which plays a vital role in follicular development (Figure 5C,D).

In external theca cells, upregulated genes exhibited enrichment in functions including “blood vessel development”, “reproductive structure development”, “response to growth factors”, “cell morphogenesis involved in differentiation”, and “cellular response to oxygen levels”. Conversely, downregulated genes in external theca cells were enriched in functions such as “blood vessel development”, “response to growth factors”, “cellular component morphogenesis”, and “negative regulation of cell population proliferation”. Notably, the functions of “blood vessel development” and “response to growth factors” were significantly enriched among both upregulated and downregulated genes. This highlights that the external theca cells play a decisive role in the dynamic maintenance of dominant and subordinate follicles and decrease external theca cell proliferation. The latter could potentially contribute to an increased incidence of follicular atresia and reduced fecundity in Du’an goats (Figure 5C,D).

The upregulated genes in vascular endothelial cells were enriched in functions such as the “negative regulation of follicle-stimulating hormone secretion”, “proton transmembrane transport”, and “cell surface receptor signaling pathway involved in cell–cell signaling”. Furthermore, downregulated genes in these cells were associated with “circulatory system processes”, “regulation of body fluid levels”, and “extracellular matrix organization”. These results further validate that low-dose FSH in Nubian goats can stimulate follicle development and ovulation, which are regulated by positive and negative feedback from the hypothalamus–pituitary axis (Figure 5C,D).

In stromal cells, upregulated genes exhibited enrichment in functions such as “reproductive structure development” and “cell chemotaxis”. This suggests that active stromal cells undergo differentiation into external theca cells, thereby facilitating the healthy development of follicles in Nubian goats (Figure 5C,D).

The upregulated genes in smooth muscle cells enriched two specific functions, namely “electron transport chain: OXPHOS system in mitochondria” and “angiogenesis”, while the downregulated genes were associated with “response to peptide hormone” and the “ovulation cycle”. These results demonstrate the provision of abundant nutrition for the development of ovarian follicles in Nubian goats (Figure 5C,D).

In immune cells, the upregulated genes exhibited enrichment in the “VEGFA-VEGFR2 signaling pathway” and the “regulation of intrinsic apoptotic signaling pathway”. Conversely, downregulated genes in the immune cells exhibited enhanced functions such as “lymphocyte activation” and a “positive regulation of leukocyte-mediated cytotoxicity”.

We further examined the expression patterns of the DEG *SERPINE2* (Figure 5E) in both polytocous and monotocous goats. A hub gene network analysis revealed that *SERPINE2* was closely associated with follicular development (Figure S2K). These results suggest that *SERPINE2* plays an essential role in facilitating the transition of follicles from dominance to successful ovulation. Altogether, our results underscore the significance of augmenting “ATP biosynthesis”, “cell proliferation”, and “cell response to hormone stimulation” to ensure the maintenance and development of dominant follicles, ultimately leading to increased ovulation rates and litter sizes in Nubian goats.

## 4. Discussion

This study presents a comprehensive single-cell transcriptomic atlas of the goat ovary during the estrus phase, elucidating gene expression profiles of distinct ovarian cell types and shedding light on the molecular mechanisms underpinning the remarkable prolificacy observed in goats. Our findings offer three noteworthy contributions. First, we elucidated gene expression signatures for 10 ovarian cell types and identified TF regulatory networks, hub genes, key pathways, and intercellular interactions. Second, we demonstrated three distinct gene expression patterns for GC subtypes and their dynamic regulatory mechanisms. Third, our analysis of prolificacy-associated alterations in gene expression revealed that the “downregulation of apoptosis”, “increased anabolism”, and “upstream responsiveness to hormonal stimulation” are associated with prolificacy. These functions are essential for the development and maturation of dominant follicles, increased ovulation in a single estrous cycle, and enhanced litter size per parity in Nubian goats. This study contributes to a deeper understanding of the molecular mechanisms underlying dominant follicle selection and the augmentation of fecundity.

The ovary is a heterogeneous and dynamic organ, and research on the characterization of ovarian cell composition and regulatory networks during specific stages of the menstrual/estrous cycle in mammals is limited. Notably, bulk-seq analysis fails to elucidate whether changes in ovarian physiology result from intrinsic molecular alterations or shifts in cell-type proportions [38]. Previous studies have generated cell atlases of the ovarian cortex in random physiological states across species, including humans [37], primates [29,39], and mice [40,41]. However, few studies have delved into the precise alterations in gene expression patterns in various ovarian cell types with regard to dizygotic births. Zengkuan Li (2021) employed scRNA-seq to reveal distinct expression profiles of GCs in ovarian follicles from goats with varying fecundity [42]. In this study, we employed snRNA-seq to successfully map the single-cell transcriptomic atlas of ovaries from polytocous and monotocous goats during the estrus phase. Our study yielded high-quality data for the in-depth analysis of gene expression patterns associated with prolificacy in ovarian cells and folliculogenesis at the single-cell level.

The cellular composition of the goat ovary was investigated, and 10 ovarian cell types were identified. Based on the unique scRNA-seq molecular signatures, the cell types and their hub gene regulatory networks were identified via WGCNA. The majority of canonical markers were confined to their respective islands, validating the robustness of our snRNA-seq approach and the reliability of our data. Although a certain proportion of ovarian germ cells were identified, conducting an accurate subtype analysis was impossible due to their small number. Therefore, spatiotemporal transcriptomic analysis of oocytes becomes imperative. Notably, GO enrichment analysis revealed significant enrichment of “the biological characteristics of sperm” in the germ cells, indicating similarities in gene expression patterns between spermatids and oocytes. This further supports the notion that the ovaries and testes may originate from a common primordial structure and represent bipotential gonads composed of multipotent somatic progenitor cells [29,43].

Multicellular organisms exhibit various cell types, each characterized by specific morphology and functions. The maintenance of these cell types relies on the orchestrated interplay between TFs and their target genes. The SCENIC algorithm can elucidate co-expression modules between TFs and their potential target genes. Employing the SCENIC algorithm, we established a network of regulons that were plausible candidates for sustaining cell-specific TF programs throughout our investigation. The results revealed several TFs specific to distinct cell types. For instance, FOXO3, NR3C1, and TEAD1 were found to be expressed in GCs, whereas vascular endothelial cells exhibited the specific expression of ELK3, ERG, and FLI1. The gene regulatory network activity scores at the single-cell level enabled the accurate identification of 10 cell types. In addition, a few TFs and their functions specific to various follicle stages or GCs were predicted, which have not been reported. These findings provide additional insights into the biological regulation of follicular development.

Currently, studies are focused on the interplay between niche and germline communication, which coordinately and reciprocally regulate folliculogenesis; however, information regarding the interactions among these signaling pathways is limited. Our study revealed the signaling pathways that synchronously and reciprocally regulate distinct cell types in the goat ovary, determining the ligands and their corresponding receptors originating from the reciprocal compartments. Initially, we analyzed the expression patterns of other components in these signaling pathways across the ovary. Additionally, we assessed heterogeneous gene expression associated with key signaling pathways, including Wnt, Notch, and TGF-β/BMP signaling pathways. Subsequently, a map of intercellular communication among each cell type was constructed. Through rigorous ligand–receptor analysis, various interacting links across the various cell types were identified. Multiple ligand–receptor pairs were identified in both follicle and niche cells, providing valuable insights into folliculogenesis regulation.

The molecular mechanisms underlying prolificacy in farm animals remain largely unknown. Here, we demonstrated that the Nubian goat’s ovarian prolificacy is linked to the cell-type-specific markers associated with the “downregulation of apoptosis”, “increase in anabolism”, and “upstream responsiveness to hormonal stimulation”. Studies have shown that a lack of functional mitochondrial aggregation can impede the follicle maturation process [44]. ATP is essential for FSH-induced GC proliferation during follicular development [45]. Furthermore, the maintenance of cell proliferation reduces follicular atresia by inhibiting apoptosis [46] and autophagy [47,48]. Previous studies have focused on the effects of hormones such as androgen, FSH [49,50], luteinizing hormone, estradiol, progesterone [51,52], anti-Müllerian hormone [53], thyroid hormones [54], and melatonin [55] on follicular development and ovulation. The findings have demonstrated the importance of hormones for follicular development. However, additional research is imperative to understand the cellular responses to hormone stimulation and utilize hormone drug dose dependence in the treatment of reproductive disorders such as POF, hypo-ovulation, and anovulation. Our findings show that *SERPINE2* plays an essential role during the transition of follicles from the dominant phase to successful ovulation. However, it should be noted that this conclusion warrants further validation. In summary, our findings provide novel insights into the genetic mechanisms underlying the prolificacy trait in goats and other mammals and elucidate potential molecular markers for enhancing prolificacy in breeding projects.

However, the current study has two limitations. Firstly, RNA techniques using single-cell suspensions lose information about the spatial relationships among cell types in target tissues; therefore, such data may be complemented by using spatial transcriptomics and/or proteomics and/or metabolomics. Secondly, although the mechanism derived in this study is based on scientific bioinformatics analytical methods, it has not been verified by molecular and cellular experiments. Further biological experiments are needed to elucidate the mechanisms behind the expression changes of these key genes and their biological functions in folliculogenesis.

## 5. Conclusions

In our study, we mapped the single-cell transcriptomic roadmap of the goat ovary and identified differences in the ovarian expression profiles between monotocous and polytocous goats. The prolificacy of goats was associated with cell-type-specific “downregulation of apoptosis”, “increased anabolism”, and “up-stream responsiveness to hormonal stimulation”. This study contributes to a broader understanding of cell identities, cell-type-specific gene signatures, and the regulatory network in the mammalian ovary. Moreover, our results provide new insights into the molecular mechanisms underlying the high reproductive rate of goats.

## Data Availability

The single-cell RNA sequencing data in this study have been deposited in the NCBI GEO database under accession code “GSE207023 (https://www.ncbi.nlm.nih.gov/geo/query/acc.cgi?acc=GSE207023; accessed on 11 February 2023)”. All other relevant data supporting the key findings of this study are available within the article and its Supplementary Materials or from the corresponding author upon reasonable request.

## Supplementary Materials

The following supporting information can be downloaded at: https://www.mdpi.com/article/10.3390/cimb46030147/s1, Table S1: Summary information of sample data identified by CellRanger; Figure S1: The cluster-to-cluster distances between cells; Figure S2: Hub network analysis of each module by WGCNA; Figure S3: Ovarian niche regulation based on ligand–receptor analysis; Figure S4: Clues for interactions between vascular endothelial cells, interstitial cells, immune cells, external theca cells, germ cells, and surrounding cells identified by CellphoneDB.
