# Supplementary material for: Unveiling the Ovarian Cell Characteristics and Molecular Mechanism of Prolificacy in Goats via Single-Nucleus Transcriptomics Data Analysis
Source: Curr Issues Mol Biol. 2024 Mar 11;46(3):2301–19. doi: 10.3390/cimb46030147 (PMC10969506; doi:10.3390/cimb46030147)
Supplement: Supplementary file 1 [file cimb-46-00147-s001.zip › cimb-2875668-supplementary.pdf]

Supplementary materials

Unveiling the Ovarian Cell Characteristics and Molecular Mechanism of Prolificacy in Goats via Single-Nucleus Transcriptomics Data Analysis

Supplementary materials

Table S1. Summary information of sample data identified by CellRanger

Items	Du'an 1	Du'an 2	Nubian 1	Nubian 2	Summary	
Estimated Number of cells	9364	10893	16988	11087	Post-Normalization Total Number of Reads	1,886,222,998
Fraction Reads in Cells	71.00%	73.10%	70.50%	75.90%	Pre-Normalization Total Number of Reads	2,494,899,963
Mean Reads per Cell	75602	46043	39585	55285	Pre-Normalization Mean Reads per Cell	51,620
Median genes per Cell	1140	1620	1321	1149	Post-Normalization Mean Reads per Cell	39,026
Number of Readers	70793619	501544385	672471623	612949336	Fraction of Reads Kept (Nubia1)	100.00%
Valid Barcodes	98.30%	98.10%	98.40%	98.40%	Fraction of Reads Kept (Nubia2)	68.40%
Valid UMIs	99.90%	99.90%	99.90%	100.00%	Fraction of Reads Kept (Duan2)	82.70%
Sequencing Saturation	85.30%	71.60%	74.50%	79.40%	Fraction of Reads Kept (Duan1)	53.60%
Q30 Bases in Barcode	95.40%	95.60%	95.50%	95.50%	Pre-Normalization Total Reads per Cell (Nubia1)	39,585
Q30 Bases in RNA Read	89.50%	91.40%	90.10%	90.80%	Pre-Normalization Total Reads per Cell (Nubia2)	55,285
Q30 Bases in UMI	94.80%	94.10%	95%	95%	Pre-Normalization Total Reads per Cell (Duan2)	46,043
Reads Mapped to Genomes	88.50%	91.70%	90.90%	92%	Pre-Normalization Total Reads per Cell (Duan1)	75,602
Reads Mapped Confidently to Genome	81.20%	84.60%	84.10%	94.80%	Pre-Normalization Confidently Mapped Barcoded Reads per Cell (Nubia1)	17,518
Reads Mapped Confidently to Intergenic Regions	14.00%	14.10%	14.60%	10.00%	Pre-Normalization Confidently Mapped Barcoded Reads per Cell (Nubia2)	25,609
Reads Mapped Confidently to Intronic Regions	0.00%	0.00%	0.00%	0.00%	Pre-Normalization Confidently Mapped Barcoded Reads per Cell (Duan2)	21,171
Reads Mapped Confidently to Exonic Regions	67.20%	70.50%	69.50%	74.80%	Pre-Normalization Confidently Mapped Barcoded Reads per Cell (Duan1)	32,682
Reads Mapped Confidently to Transcriptome	62.00%	64.00%	63.80%	62.40%	Estimated Number of Cells	434,988

Reads Mapped Antisense to Gene	1.60%	1.90%	2.00%	2.10%	Fraction Reads in Cells	72.40%
Total Genes Detected	20029	20710	20813	20775	Median Genes per Cell	1,263
Median UMI Counts per Cell	1926	3383	2400	2011	Median UMI Counts per Cell	2290

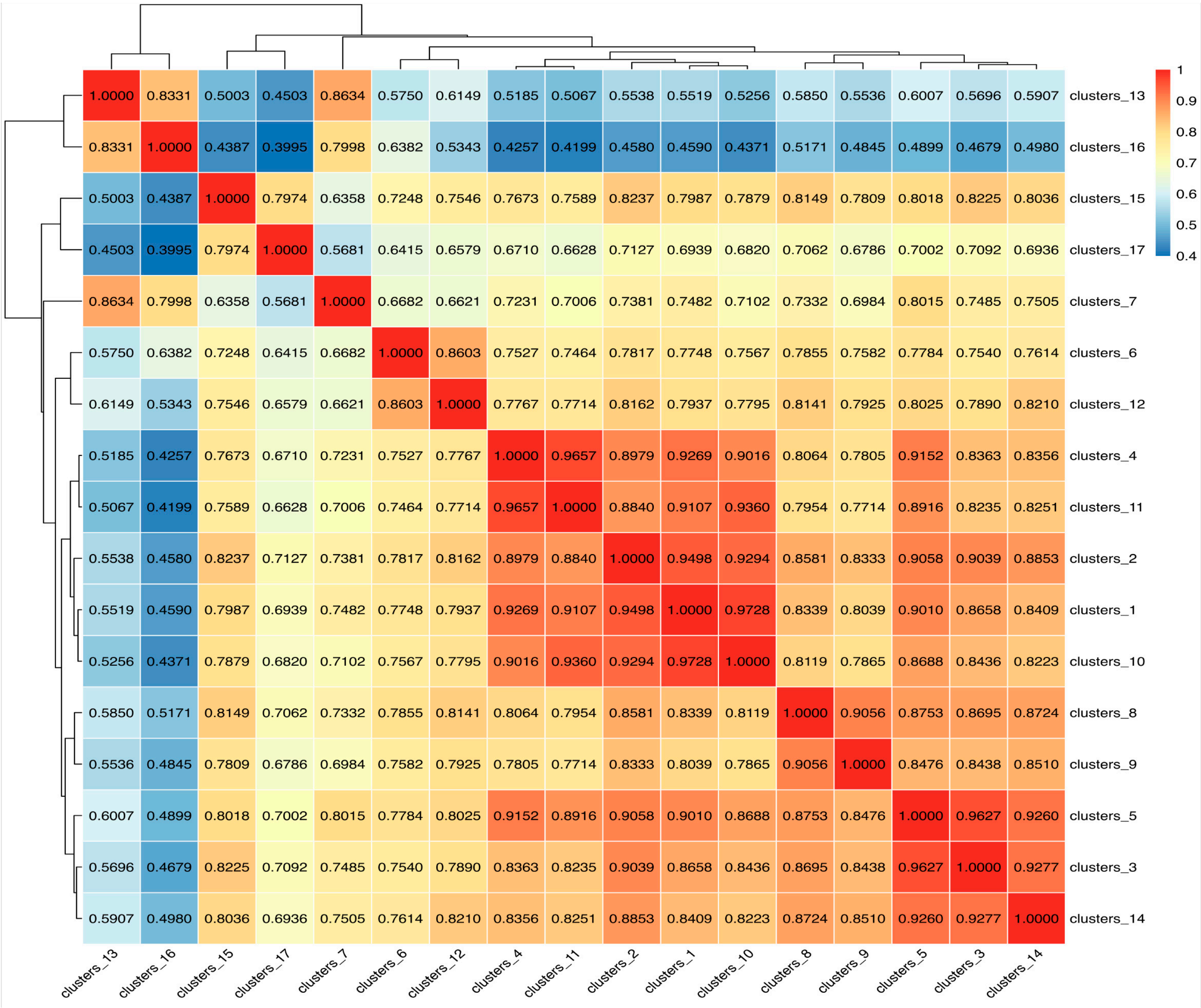

Figure S1. The cluster-to-cluster distance between the cells.

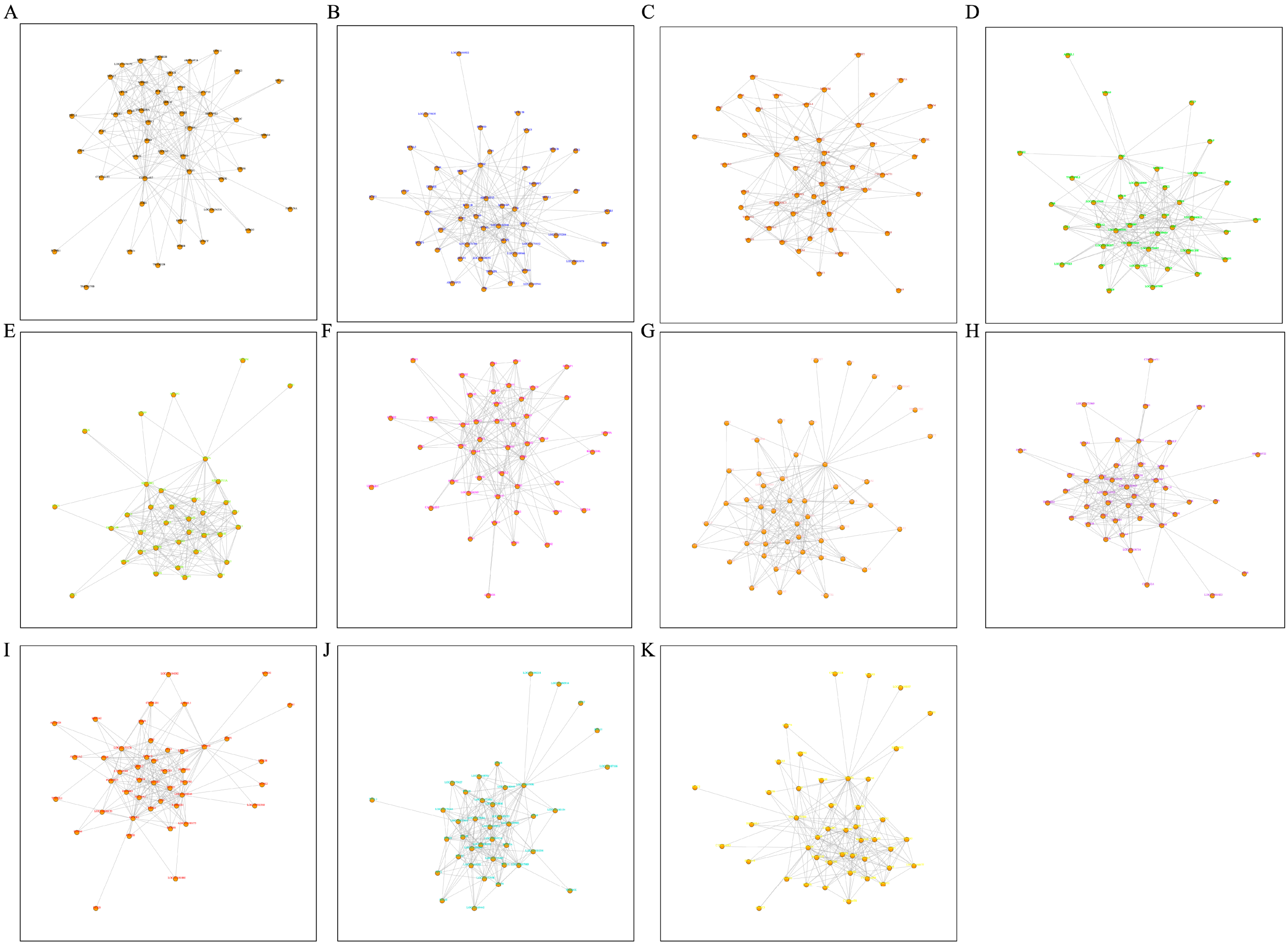

Figure S2. Hub network analysis of each module by WGCNA. **(a)** Black hub network—GCs. **(b)** Blue hub network—B cells. **(c)** Brown hub network— vascular endothelial cells. **(d)** Green hub network—Nature killer T cells. **(e)** Green-yellow hub network—Internal theca cells. **(f)** Magenta hub network—smooth muscle cells. **(g)** Pink hub network—Smooth muscle. **(h)** Purple hub network—Macrophage. **(i)** Red hub network—Stromal cells. **(j)** Turquoise hub network—External theca cells. **(k)** Yellow hub network—Germ cells.

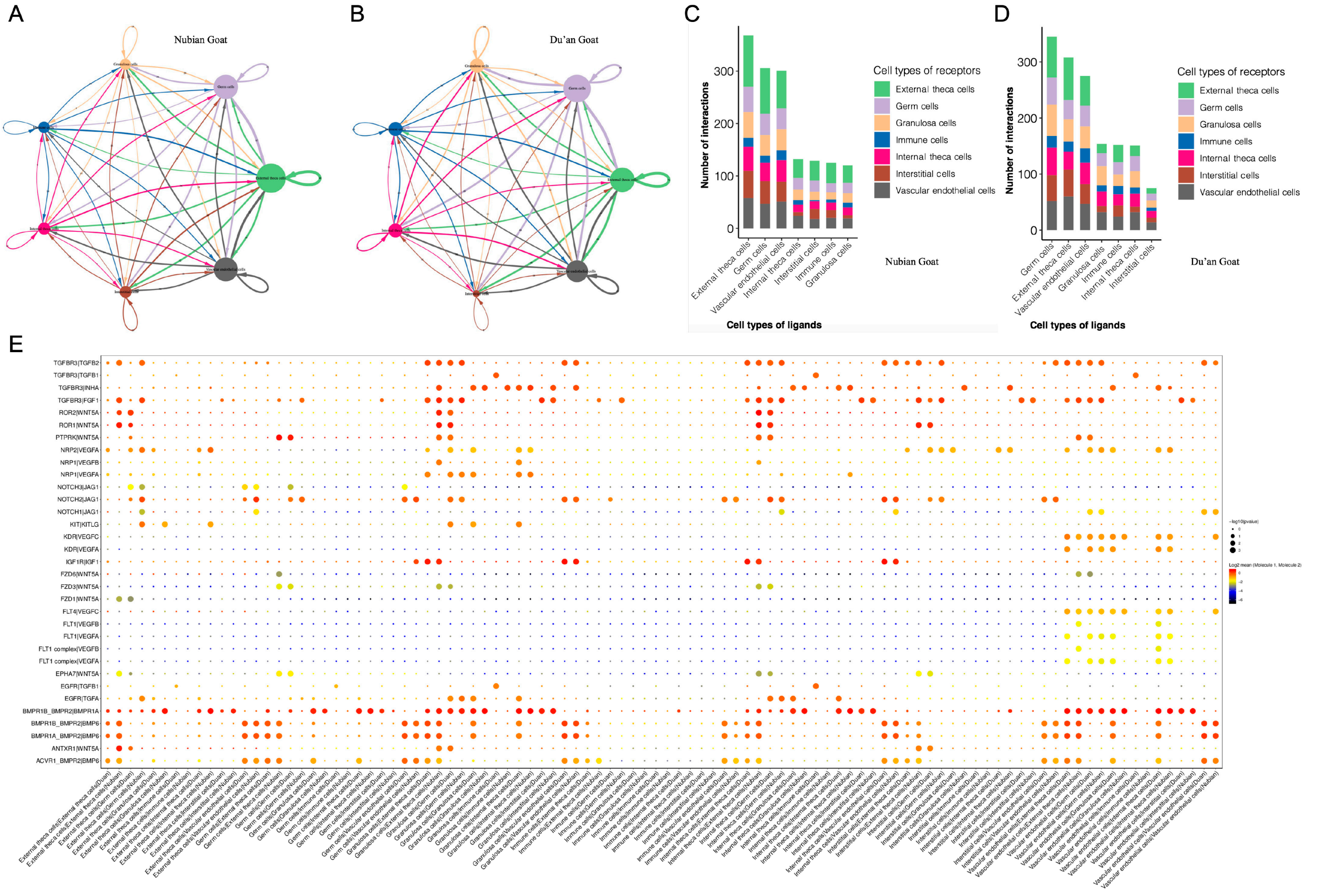

Figure S3. Ovarian niche regulation based on ligand-receptor analysis. (a-b) Network plots show the ligand-receptor interaction in each type and their niche cells in Nubian (a) and Du'an goats (b). The connected line indicates cell-cell communication. The thickness of the lines and the size of the nodes are positively correlated with the number of ligand-receptor interaction events. (c-d) Quantifying cell-cell communication frequencies in each cell type based on putative ligand-receptor interactions in Nubian (c) and Du'an goats (d). E Expressional patterns of key signaling pathway components in each cell type and their niche cells in Nubian and Du'an goats, including expression of genes linked to *Wnt*, *VEGF*, *Notch*, and *TGF-β/BMP* signaling pathways.

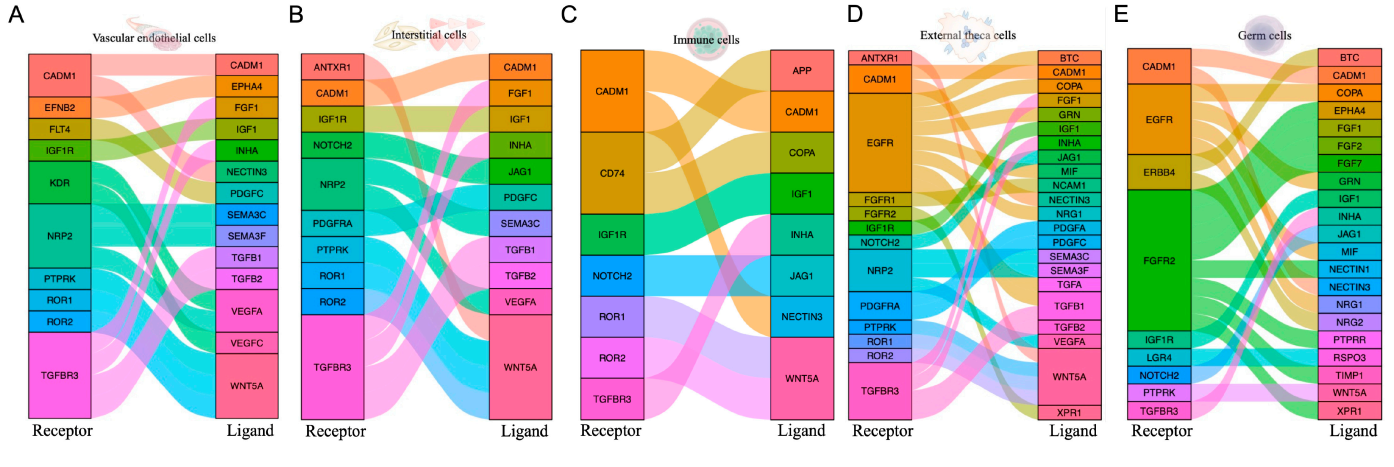

Figure S4. Clues for interactions between vascular endothelial cells (a), interstitial cells (b), Immune cells (c), external theca cells (d), germ cells (e), and surrounding cells identified by CellphoneDB.
